# Highly stretchable and sensitive silicone composites with positive piezoconductivity using nickel powder and ionic liquid

**DOI:** 10.1063/5.0124959

**Published:** 2023-02-03

**Authors:** R. Matsuda, Y. Isano, K. Ueno, H. Ota

**Affiliations:** 1 Department of Mechanical Engineering, Yokohama National University, 79-5 Tokiwadai, Hodogaya-ku, Yokohama, Kanagawa 240-8501, Japan; 2 Department of Chemistry and Life Science, Yokohama National University, 79-5 Tokiwadai, Hodogaya-ku, Yokohama, Kanagawa 240-8501, Japan; 3 Center for Advanced Chemical Energy Research Center, Institute of Advanced Sciences, Yokohama National University, 79-5 Tokiwadai, Hodogaya-ku, Yokohama, Kanagawa 240-8501, Japan; 4 Graduate School of System Integration, Yokohama National University, 79-5 Tokiwadai, Hodogaya-ku, Yokohama, Kanagawa 240-8501, Japan

## Abstract

Conductive rubber composites are mixtures of stretchable rubber and conductive materials. They can achieve conductivity and high elasticity and are used in soft robots and wearable devices. In general, these composites exhibit high electrical resistance owing to their bonds between the fillers breaking during elongation. However, there are several types of composite materials that decrease resistance by increasing contact between the conductive materials during elongation through optimization of the shape and size of the filler. These composite materials can rapidly decrease the resistance and are expected to be applicable to switch in electric circuits and sensors. However, to use such composite materials in circuits, the electrical resistance at the time of resistance reduction must be sufficiently low to not affect the electric circuit. To achieve this, a considerable amount of filler must be mixed; however, this reduces the elasticity of the composite. Simultaneously achieving elasticity of the composite and a sufficient decrease in the resistance is challenging. This study developed a conductive rubber composite gel by mixing silicone rubber, ionic liquid, and metal filler. Consequently, the composite achieved an elongation rate of over six times and a decrease in the resistance of less than 1/10^5^. In addition, this composite material was used as a switch circuit wherein an electric circuit is turned on and off according to elongation through a connection to a DC power source.

## I. INTRODUCTION

Conductive rubber composites are mixtures of stretchable rubber and conductive materials and have been extensively studied. Silicone rubber^1–3^ and fluororubber^4^ are frequently used as rubber materials, whereas carbon nanotubes,^5^ graphene,^6^ silver nanowires,^7,8^ and silver flakes^1,4,9^ are used as conductive materials. The conductive composites mixed with such materials exhibit high elasticity owing to their basal material being rubber. Therefore, these composites are used as wiring, sensors, and switches in soft robots and wearable devices with electric circuits.

In general, composites realize conductivity through electron transfer between conductive fillers. Their properties vary depending on the size, volume ratio, shape of the fillers, and the bonding method (with or without sintering) between the fillers. Various composites have been proposed, such as those that increase resistance in response to tensile deformation using carbon nanotubes^5^ or silver nanowires^7,8,10^ and those that decrease resistance in response to strain using liquid metal and iron particles^2^ and Ni particles.^11^ Thus, composites containing conductive fillers can control electrical properties by optimizing the type of filler.

Typically, the bonds between fillers in such composites are broken through tensile deformation, which opens gaps between fillers and reduces electrical conductivity. In a previous study using sintered silver nanowires,^7^ the bonds between the nanowires broke during elongation, and the conductivity was reduced. In another study using silver flakes,^12^ the main rubber material itself broke during elongation, and the conductivity was decreased owing to the bonds between the fillers being broken. Such materials are used as strain sensors by primarily reading the change in the resistance and provide feedback in soft robots and wearable devices. In contrast, research has also been reported on increasing conductivity depending on strain. This type of composites increases conductivity by increasing the bonding between fillers during elongation. Composites using micro-ordered nickel powder of a spike-shaped surface^11^ experience a sharp decrease in the resistance during elongation, compression, and torsion. In addition, those wherein micro-ordered iron particles and liquid metal are encapsulated in silicone rubber^2^ can result in the rapid development of conductivity by increasing the contact between the liquid metal and iron particles during elongation. Such composites can be used as sensors by utilizing resistance change, as in the past, and as switches in circuits to exploit the rapid and sharp drop in conductivity.

However, to use the composites that increase conductivity during elongation for these applications, sufficient conductivity that does not interfere with the operation of the circuit with a decrease in the resistance must be obtained. Consequently, a considerable amount of conductive filler must be mixed; however, in the case of rubber materials, this results in a significant reduction in elasticity. For example, the composite proposed in the study by Bloor *et al.*^11^ shows a drop in the resistance of up to 10^11^ times in tension but only about 40% elongation. In the study by Yun *et al.*,^2^ compositions with elongation rates of 150% or more are presented, but their resistivity does not decrease to a value that does not interfere with the operation of electric circuits. On the other hand, compositions with a high resistance reduction rate are presented, but the maximum strain is about 30%. Therefore, when considering applications to soft robots, wearable devices, and other devices that require high elongation, both high elongation and rapid changes in conductivity must be realized.

Based on these backgrounds, this study proposes a new positive-piezoconductive rubber composite that exhibits a sharp drop in the resistance in tension and is 20 times more extensible than existing positive-piezoconductive materials. The conductive rubber composite is made by mixing a rubber material with a metal and an ionic liquid. The proposed composite showed 1 × 10^5^ times increase in conductivity with elongation with a maximum elongation rate of 600%. Silicone rubber and ionic liquid can form a gel state and increase its elasticity, because the nonvolatile ionic liquid stays inside the rubber in a liquid state. Furthermore, a conductive composite was made by mixing a 1.5–2 times the mass ratio of nickel powder to silicone rubber. This composite could be stretched to approximately six times its original length at maximum elongation, and its conductivity was 1 × 10^5^ times higher than that in the unstretched state. The study further demonstrated wearable devices and soft robots using the proposed composite.

## II. RESULTS

**FIG. 1.**
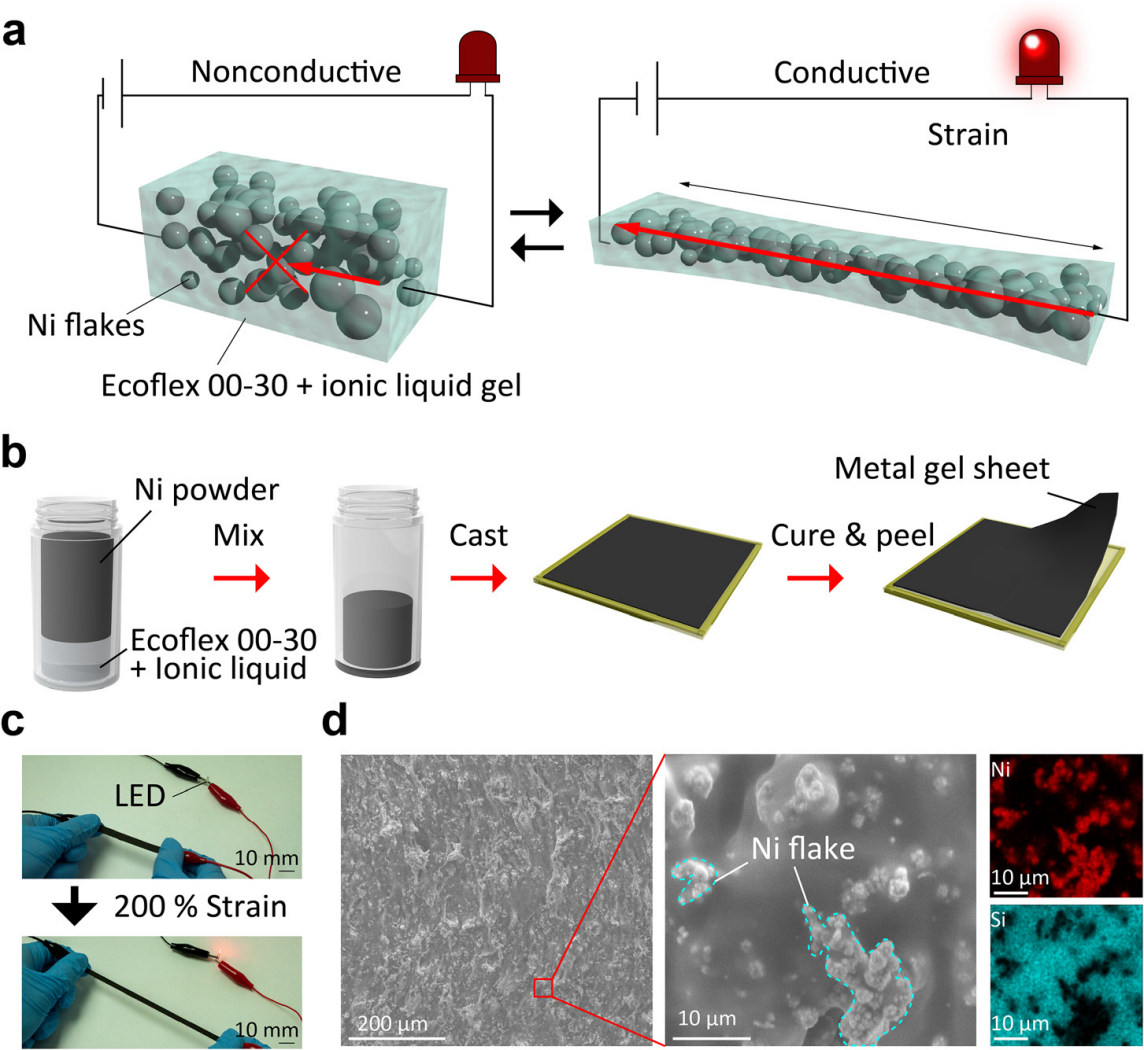
Concept of the highly stretchable conductive composite. (a) Schematic of the composite produced. Strain deformation causes the metal powders in the composite to bond together, and conductivity appears during tension. (b) Material fabrication method. This composite can be made by simply mixing Ecoflex, nickel powder, and ionic liquid. (c) LED lighting demonstration. The conductivity is exposed during tension, and the LED is turned on. (d) SEM and EDS images of the composite. Nickel powder is dispersed in the composite.

A conceptual image of the composite resin prepared in this study is shown in Fig. 1(a). The proposed composite was composed of nickel flake, silicone resin (Ecoflex 00–30), and ionic liquid [N-Methyl-N-propylpyrrolidinium bis(trifluoromethanesulfonyl)imide]. The composite resin was conductive or insulating depending on whether it was elongated or not, respectively. This composite contains ionic liquid. The ionic liquid is a nonvolatile liquid material, which was retained as a liquid inside the resin. Consequently, the cross-linking between the silicone resins was more stretchable, and the composite's elongation rate was increased.

The fabrication method of this composite is shown in Fig. 1(b). It can be fabricated via simple mixing and curing of silicone resin, nickel flake, and ionic liquid. In the uncured state, the composite has a putty-like appearance; thus, it can be cast and cured into various shapes. Fig. 1(c) demonstrates the stretching of the composite. As shown, the current flowed only when the composite was stretched, and the LED was turned on. As shown in Fig. 1(d), the scanning electron microscope (SEM) and energy dispersive x-ray spectrometer (EDS) images indicated that the nickel powder was dispersed in the resin. In addition, the particle diameter of the Ni powder ranged from 5 to 20 *μ*m (Fig. S1).

**FIG. 2.**
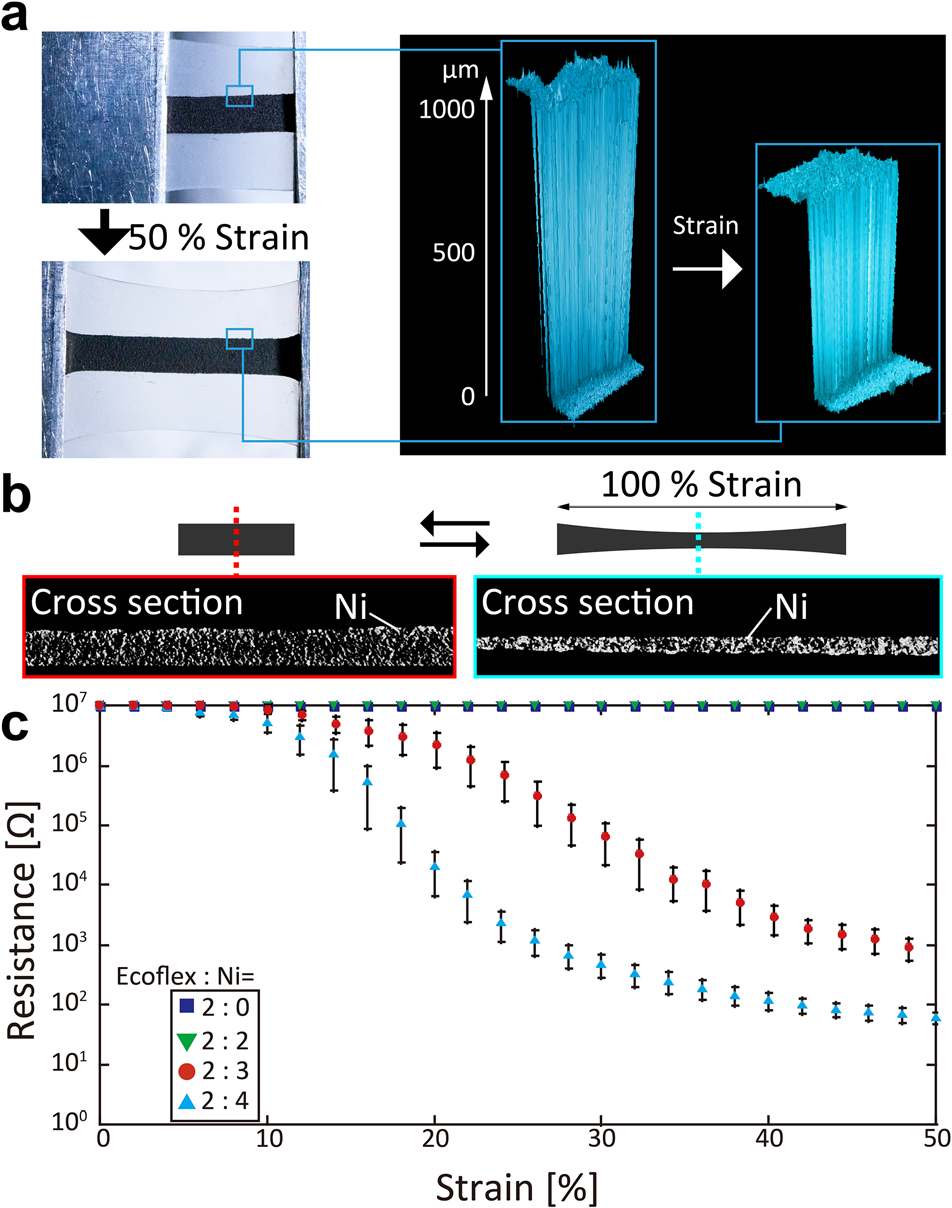
Electrical properties of the composite. (a) Thickness change during tensile deformation as measured by laser microscopy. When 50% lateral strain was applied, the thickness decreased to approximately 70%. (b) CT image of a composite section. The nickel powder is shown in white. The distance between the powders decreased when subjected to 100% strain in the horizontal direction. (c) Electrical resistance to tensile deformation. The electrical resistance was measured for different amount ratios of nickel powder under strain up to 50%. Error ranges indicate standard errors (n = 5).

The electrical properties of the composite under strain were investigated, as shown in Fig. 2. The thickness change of the composite was measured via a laser microscope under 50% strain. The results indicated that when subjected to 50% horizontal strain, the thickness of the composite was approximately 70%. Furthermore, as shown in the computed tomography (CT) scan image of Ni powder dispersed in the composite [Fig. 2(b)], the Ni powder observed in white was vertically compressed owing to horizontal distortion, which caused the Ni powder to agglomerate, and the contact area between the powders increased. Thereafter, CT images were also obtained for a cross section horizontal to the strain direction (Fig. S2). Evidently, the density of Ni powder, which is detected in white, increases during elongation.

A Ni content was changed to 0, 2, 3, and 4 with the mass ratio of Ecoflex: ionic liquid = 2:0.1, and the change in the electrical resistance at 50% strain was investigated [Fig. 2(c)]. As the composite functioned as an insulator when not stretched, a fixed 10 MΩ resistor was connected in parallel to ensure conductivity in the insulating state (Fig. S3). Fig. 2(c) shows that for a Ni powder 2:2 weight ratio for Ecoflex or less, no conductivity was exhibited even at 50% strain. However, with Ecoflex: Ni = 2:3 weight ratio, the resistance began to drop at approximately 12% strain and further dropped to approximately 100 Ω at 40% strain. This is less than 1/10^6^ of the resistance of the insulating state. In the case of the composite with Ecoflex: Ni = 2:4 weight ratio, the resistance drop was earlier. The resistance began to decrease at approximately 4% strain and reduced to 100 Ω at approximately 30% strain. This indicates the resistance drop point can be changed depending on the amount ratio of Ni powder. The tensile test was repeated 100 times on the composite (Fig. S4). The resistance value could be reduced even after 100 tensile test cycles were performed. However, the decrease in resistance with tensile strain became progressively smaller, and finally, the resistance increased to approximately 1 MΩ at 50% strain.

The changes in electrical properties of the Ecoflex–Ni composites due to the addition of ionic liquids are compared and shown in Fig. S5. It shows that the addition of ionic liquids decreased the strain at which the resistance begins to drop as well as the lower limit of resistance.

A similar verification was performed with a stretchable silicone conductive composite with sintered silver. This composite showed that the resistance increased in response to strain (Fig. S6). The surface SEM image of the sintered silver composite was acquired in both the unstretched and stretched conditions (Fig. S7). The images show that the sintered silver composite formed a smooth silver film on the surface when not stretched, whereas cracks appeared in the silver film when stretched. In addition, SEM images of the composite fabricated in this study were acquired under nonstrain and strain conditions (Fig. S8). The silicone–Ni–ionic liquid composite material that we fabricated did not appear to crack even at 100% elongation.

**FIG. 3.**
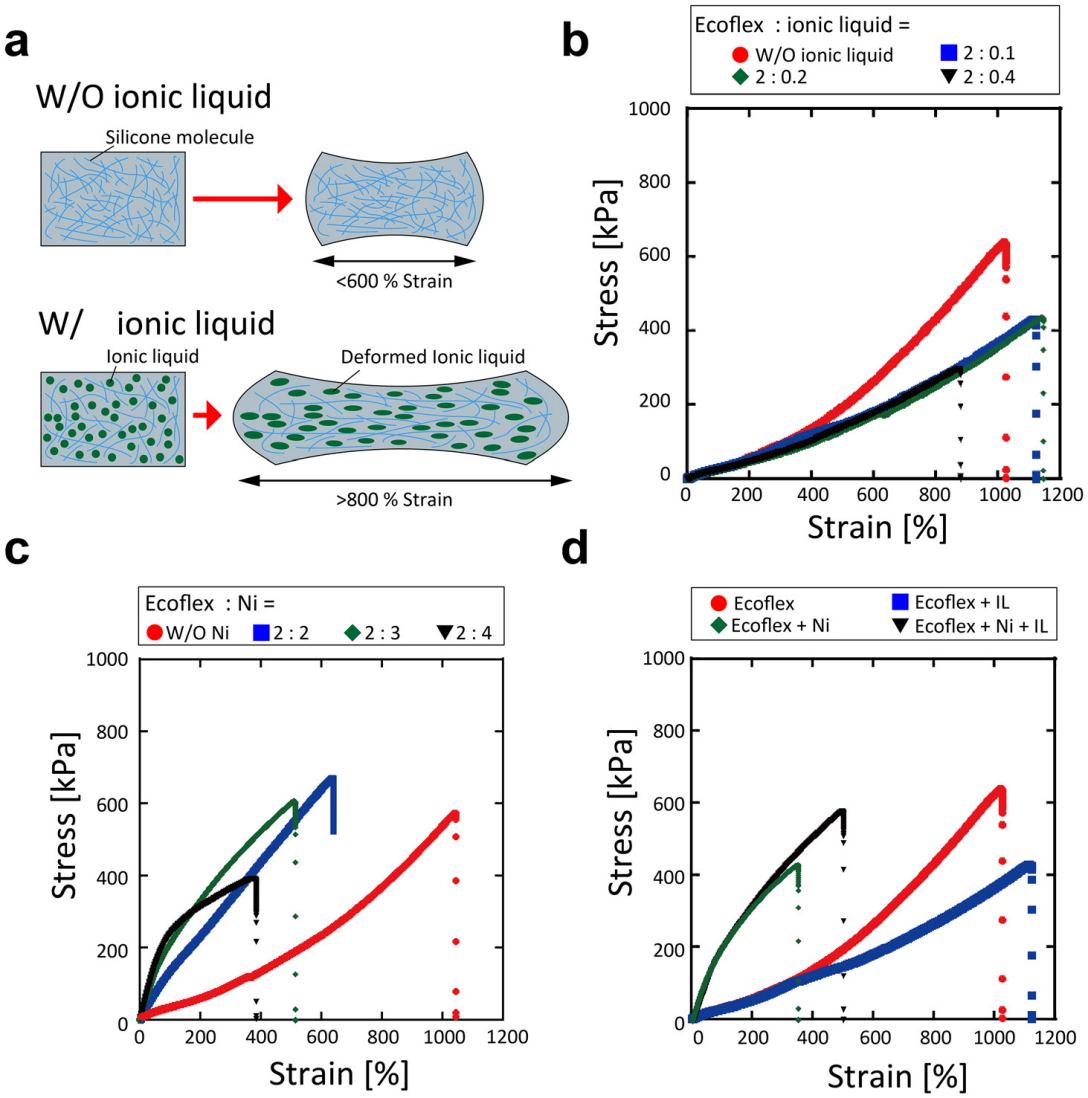
Mechanical properties of the composites. (a) Schematic of silicone rubber elasticity increased by mixing ionic liquid. The ionic liquid remains in the liquid form in the rubber; the stretchability of the rubber material increases. (b) Variation in elasticity with ionic liquid volume ratio. With an increase in the amount of ionic liquid, the rubber becomes more flexible and elasticity increases. Maximum elongation decreases as the amount of ionic liquid exceeds a certain ratio. (c) Change in stretchability owing to mixing of nickel powder. Stretchability of the composite decreases with further mixing of nickel powder. (d) Elasticity with and without ionic liquids (IL) and nickel powder. Mixing nickel powder reduces the stretchability of the composite, whereas mixing ionic liquids makes the composite maintain stretchability of approximately 500% even with large amounts of nickel.

The mechanical properties of the proposed composite were tested, as shown in Fig. 3. The ionic liquid was nonvolatile; thus, it was dispersed in silicone in the liquid form. As the liquid state ionic liquid can be easily deformed in response to elongation, the composite exhibited high elasticity [Fig. 3(a)]. The stress–strain diagrams were measured by varying the amount of ionic liquid mixed with silicone resin without Ni powder to 0, 0.05, 0.1, and 0.2 weight ratios to Ecoflex [Fig. 3(b)]. Five tensile tests were conducted; Young's modulus was determined from the tensile range up to 3% and is shown in Table S1. No significant change in Young's modulus was observed with the addition of ionic liquid. On the other hand, Young's modulus increased with an increase in the amount of Ni powder added.

The average breaking strain is shown in Fig. S9. Fig. S9(a) shows that the addition of ionic liquid improved the maximum elongation length. However, the maximum elongation length decreased when the ionic liquid amount exceeded 0.4 weight ratio. The stress–strain diagrams were compared for W/and W/O Ni powder and ionic liquid.

Tensile tests were performed on composites with 0.1 of ionic liquid and 0, 2, 3, or 4 of Ni powder added to 2 Ecoflex by weight ratio. The result in Fig. 3(c) and S9(b) shows that the slope of the stress–strain diagram increased with an increase in the amount of Ni powder, whereas the elongation length decreased. As evident from Fig. 3(d), the Ni powder mixture decreased the elongation at break and increased its hardness, whereas the ionic liquid mixture improved the composite's stretchability.

**FIG. 4.**
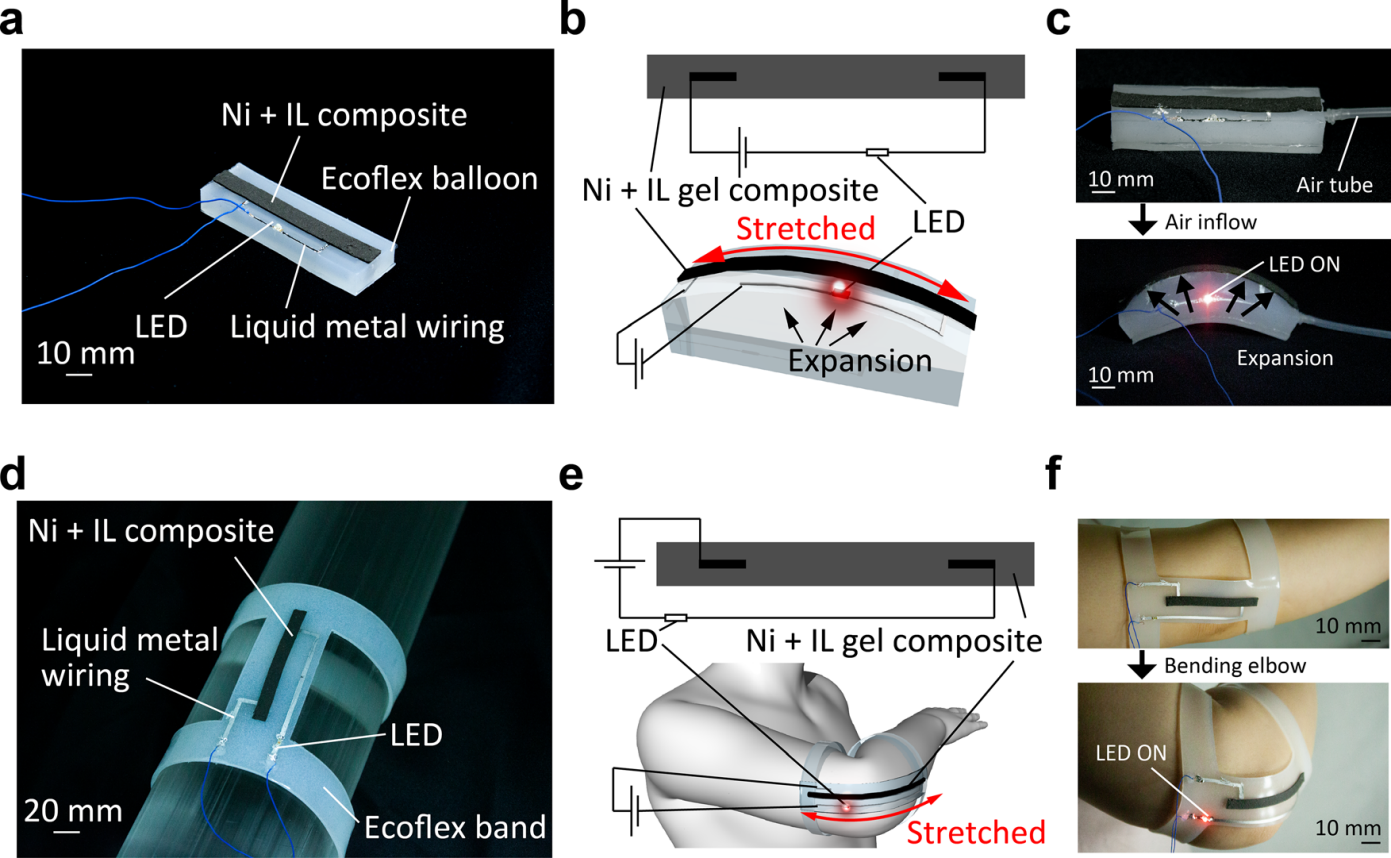
Demonstration of the composite. (a) Balloon-type soft actuator device. (b) Circuit overview diagram. (c) Photograph during device operation. The composite is stretched as the balloon expands, thus exposing its conductive properties and lighting the LED. (d) Wearable device capable of detecting elbow bending and elongation. (e) Circuit overview diagram. (f) Photograph of the device attached to the elbow. Bending the elbow stretches the composite, and the LED light is turned on.

Demonstration devices were fabricated using the proposed composite. The composite sheet and red LED were connected to the power supply and attached to the balloon actuator [Fig. 4(a)]. The fabrication method of this device is shown in Fig. S10. The circuit was fabricated by patterning a mixture of liquid metal and Ni powder^13^ as the expansion wiring. As shown in Fig. 4(b), the expansion of the balloon actuator caused the composite sheet to stretch and the composite became conductive. Subsequently, power was supplied to the LEDs, causing them to light up. Fig. 4(c) shows a photograph of the actuator in operation. When the balloon was inflated by injecting air through the tube connected to the balloon, the composite sheet was inflated. Only upon inflation did it become conductive, and the LEDs lit up. However, when the air was removed, the composite sheet became nonconductive and the LED turned off. A similar sensor was also used as a wearable sensor [Fig. 4(d)]. The fabrication method of this device is shown in Fig. S11. This device can be worn on the elbow to detect elbow bending and turn on an LED when the elbow bends [Fig. 4(e)]. Biocompatible Ecoflex and Galinstan were used for the device, and the structure was optimized so that the composite containing ionic liquids does not adhere to the skin. The bending of the elbow caused the composite sheet to stretch and the LED to light up. When the elbow was further extended, the composite material was insulated again and the LED turned off [Fig. 4(f)]. The change in the resistance of the composite due to elbow bending is shown in Fig. S12.

## III. DISCUSSION

Fig. 2(a) and 2(b) show that the composite prepared in this study shrunk in both width and thickness during elongation. In composites composed of metallic powder and nonconductive rubber material, the conductivity rapidly occurred when the volume ratio of metallic powder to rubber material exceeded a certain percentage.^14^ This is because metal powder above a certain volume ratio is essential in the composite to facilitate establishment of electrical connections as a circuit.^15^ In the composite fabricated in this study, when tension occurred, the rubber material, Ecoflex, was compressed and its cross-sectional area reduced. Subsequently, the density of nickel in the rubber material increased rapidly, which allowed the rubber material to be energized. In previous studies on composites with silica filler dispersed within raw rubber, the raw rubber was retained inside the aggregates of silica filler.^16,17^ The distance between silica fillers increased rapidly owing to the leakage of raw rubber inside the aggregates and the collapse of the aggregates when they were pulled. Similarly in this study, the increase in the volume ratio of Ni powder owing to the compressive change of the rubber material and the collapse of the aggregates changed the connection structure between the powders. The Ni powder particles connected with each other at more points, which may be the cause of a significant change in conductivity [Fig. 2(b)].

The resistance of the composite was more than 10 MΩ when not extended, whereas it reduces to approximately 10 Ω when extended [Fig. 2(c)]. This is a resistance drop of more than 1/10^5^, which is a considerably larger drop ratio than in previous studies.^2,18^ The elongation ratio at which the resistance drop begins can be controlled according to the mass of Ni powder contained inside. In addition, the resistance drop started at approximately 12% in the case of 1.5 times the weight of Ni powder relative to Ecoflex, and at approximately 4% in the case of two times the weight of Ni powder. However, when three times the weight of Ni powder was added to an equal amount of Ecoflex, the composite did not harden and formed a polymer structure. Therefore, further optimization of the base rubber material and ionic liquid was required to cause a drop in the resistance at an elongation rate of less than 4%. Moreover, when the amount of Ni powder was reduced and only equal weight to Ecoflex was added, no conductivity was observed even at elongation rates above 50%. This can be improved by optimizing the rubber material, ionic liquid, and particle size and shape of the Ni powder, and by using it in combination with other materials.

Figure S4 shows the change in the resistance over 100 tensile tests. This can be attributed to the plastic deformation of the silicone–Ni–ionic liquid composite. The composite is plastically deformed by repeated tensile tests, resulting in overall elongation, and the amount of strain at the end of the cycles could be smaller than the amount of strain at the beginning of the cycles. Therefore, suppression of plastic deformation by the addition of liquid metal or other means may contribute to improved cyclic stability.

Figure S5 shows that the lower limit of resistance decreased with the addition of ionic liquids in addition to the decrease in the strain at the beginning of the resistance drop. On the other hand, ionic conduction associated with migration of ionic liquids was not observed. This phenomenon could be attributed to addition of ionic liquids, which swelled Ecoflex, and the resulting decrease in cross-link density, which increased the mobility of Ni particles within the composite.

The composite fabricated in this study exhibited enhanced elasticity owing to the addition of ionic liquid to Ecoflex. The nonvolatile nature of the ionic liquid enabled it to remain in a liquid state inside the rubber, which replaced the cross-linking of Ecoflex and improved its elasticity [Fig. 3(a)]. Many studies have been conducted on gelation of polymers through the addition of ionic liquids, including studies on improving flexibility by mixing ionic liquids with urethane polymers and polyvinyl chloride.^19^ This can enable applications such as wearable devices and actuators that exploit the high flexibility unique to ion gels. When 0.1 weight ratio of ionic liquid to 2 Ecoflex was mixed, the elongation at break exceeded 1100% [Fig. 3(b)], which is more than 1.1 times than that without ionic liquid. Simultaneously, the slope angle of the stress–strain diagram decreased, indicating that the hardness decreased as well. Hydrogels with a high elongation ratio of more than 20 times have been proposed;^20^ however, they are not suitable for long-term service because they dry and harden with time. The composite fabricated in this study was primarily composed of ionic liquids and silicone resin; hence, its properties are not likely to changeover time. However, the addition of excessive amounts of ionic liquid reduces the elongation at break in exchange for a further decrease in hardness; when more than 0.2 weight ratio of ionic liquid to 2 Ecoflex was mixed, the hardness decreased, whereas the maximum elongation length was below 800%. This indicates that excess ionic liquid in the silicone resin results in a decrease in the elongation length. This may attributed to the excess ionic liquid inhibiting cross-linking between the silicone resins, causing them to change to a more liquid state. Consequently, their robustness to tensile forces is reduced.

The addition of conductive powder to ion gels can add electrical functionality and enable their use as conductive electrodes or wiring materials. However, when conductive powders are mixed into the polymer, the hardness increases, and the elongation rate decreases [Fig. 3(c)]. This phenomenon is also observed when other conductive particles such as carbon powder, carbon nanotubes, copper powder, silver nanoparticles, and silver flakes are mixed, and may be because it is more difficult for solid powders to change shape compared to rubber materials. Moreover, the decrease in elongation and the increase in hardness depend on the ratio of the added particles: the strain at break was more than 1000% without Ni powder, whereas that at break dropped to 600%, 550%, and 400% with an increase in the amount of Ni powder (2, 3, and 4 weight ratios to Ecoflex, respectively). In contrast, the maximum strain at break of the flexible conductive composites prepared via the addition of ionic liquids and carbon nanotubes to KE-441 (silicone resin similar to Ecoflex used in this study) was approximately 200%. This indicates that the strain at break is highly dependent on the physical properties of the main ingredient silicone in addition to the amount of ionic liquid added and the type of metal powder.

Furthermore, as shown in Fig. 3(c), the stress–strain diagram of the powder-mixed composite showed a change in the direction of inclination at the beginning of elongation compared to the unmixed composite. This can be attributed to the decrease in hardness because of the disintegration of the aggregates. Studies using silica and raw rubber^21^ have reported that the formation and strength of the aggregates affect the hardness of the entire composite. Therefore, this mechanical property can be optimized by selecting the type of filler, particle size, and aggregate size, which affect aggregate formation and strength. In this study, the addition of metallic powder to silicone rubber increased hardness and significantly reduced elongation. Moreover, the addition of ionic liquid imparted electrical properties while maintaining elongation [Fig. 3(d)].

Accordingly, Table I presents a comparison of conductive composites composed of flexible materials. The composite fabricated in this study achieved a sharp drop in the resistance with tensile deformation and high elasticity.

**TABLE I.** Comparison with other previous studies.

Author	Filler material	Resistance changes under strain	Max strain
Kim *et al.*^22^	Single wall carbon nanotube	100% Increase	200%
Yoon *et al.*^12^	Ag flake	100%–3000% Increase	300%
Kim *et al.*^1^	Ag flake	16000% Increase	1800%
Nakanishi *et al.*^9^	Ag flake	200% Increase	45%
Bloor *et al.*^11^	Ni powder	1/10^14^ Decrease	38%
Hong *et al.*^18^	Multi-wall carbon nanotube	75% Decrease	100%
This work	Ni powder	Over 1/10^5^ decrease	600%

Finally, this study demonstrated two devices of a soft robot and wearable device. Many studies have been conducted to mount sensors on soft actuators and stretchable wearable devices; however, they must have high deformability in response to the deformation of the underlying device. In these studies, when a finger, arm, or actuator drive unit is bent or extended, a sensor laid on its top surface is extended, and feedback is obtained by reading the change in the electrical resistance that occurs during the extension. However, to use such a sensor to facilitate turning on and off electrical components such as LEDs, an additional external circuit is required for conventional sensors whose resistance increases in the case of extensions. The composite sensor fabricated in this study addressed this problem by using the rapid resistance drop owing to extension to switch the current on and off directly using only a single series circuit, as shown in Figs. 4(b) and 4(e), without the need for an external circuit. Figure S12 shows the change in resistance of the composite with elbow bending angle. Although there was an error range, the resistance decayed to less than 1% of its initial value with bending around 120°. It can be possible to control the angle at which the LED lights up by optimizing the length and placement of the composite. However, this composite is suitable for use only as a digital read-like sensor because the resistance drop is excessively steep, and reading out minute angle changes as a continuous value is challenging. In addition, it is desirable to avoid adhesion between the composite and the skin by optimizing the structure when using the composite in wearable devices, even though the composite is gelatinized and the ionic liquid does not leak out.

## IV. CONCLUSION

This study formed a stretchable conductive composite using silicone rubber and ionic liquid, followed by mixing a considerable amount of metallic powder into the rubber material. The proposed composite exhibited two characteristics: it can be extended up to six times its initial length, and its electrical resistance decreased significantly when subjected to tensile deformation. The composite fabricated in this study can be used as a sensor or switch on an electronic device that undergoes large deformation and stretching by exploiting these properties. Furthermore, it can be applied to feedback circuits for flexible actuators, artificial skin devices mounted on the skin, and medical devices that require close contact with the body.

## V. METHODS

### A. Ni microflake composite metal gel fabrication

First, silicone rubber Ecoflex 00–30 agent A, agent B, ionic liquid [N-Methyl-N-propylpyrrolidinium bis(trifluoromethanesulfonyl)imide, Kanto chemical], and Ni microflake powder (<50 *μ*m, Merck) was added in a vial bottle, at a mass ratio of 5:5:0.5:15. Next, the uncured Ecoflex, ionic liquid, and Ni powder were mixed by using a planetary centrifugal mixer (Thinky) for 2 min at 2000 rpm. Then the composite liquid was poured into the mold made with a 3D printer and cured for 20 min at 70 °C. After curing, the metal gel sheet was peeled off from the mold. Thus, the Ni micro flake silicone ion–gel composite sheet was obtained.

### B. Demonstration devices fabrication

#### 1. Material preparation

The balloon type demonstration device was made of two different silicone resins with different hardnesses. The softer resin is Ecoflex 00–30, and the harder resin is a mixture of Ecoflex 00–30 and KE-1606 at a mass ratio of 1:1. In addition, the electrodes and wiring on the balloon surface were made of liquid metal and Ni powder composite paste. This paste was prepared by the following method. First, Galinstan and Ni powder was added into a vial bottle at 15:0.9 weight ratio. Next, this composite was mixed using a planetary centrifugal mixer for 20 min at 2000 rpm. Then the composite was dispersed using an ultrasonic prober for 112 s. The total process energy was 6.1 kJ. Finally, this composite was left to stand overnight to obtain the liquid metal–Ni composite paste.

#### 2. Balloon actuator type demonstration

The fabrication method of the balloon type LED demonstration device is shown in Fig. S8. First, the upper mold was fabricated with a 3D printer (i). Ecoflex 00–30 was poured into the mold (ii), then cured and peeled (iii). As follows, the lower mold was fabricated with the 3D printer (iv) and the mixture liquid of KE-1606 and Ecoflex 00–30 was poured into it (v). After curing, the harder resin sheet was peeled off from mold (vi). These two parts were bonded using Sil-poxy (vii), and the balloon actuator was fabricated. Subsequently, polyimide film mask was put on the balloon (viii), and the Ni–ionic liquid paste was applied onto the top surface of the balloon (ix). Finally, the Ni metal gel sheet and LED were stick on the balloon(x).

#### 3. Wearable device type demonstration

The fabrication method of the wearable elbow angle detection sensing device is shown in Fig. S9. First, a mold was fabricated using a 3D printer (i), and the Ecoflex 00–50 was poured into it (ii). After curing, the sheet was peeled off from the mold (iii). This part is a band for wearing on the arm. Next, a polyimide film mask was put on this band (iv), and Ni–liquid metal paste was applied onto the surface (v). Finally, the Ni metal gel sheet and LED were applied on the band, and the wearable sensing device was obtained (vi).

## SUPPLEMENTARY MATERIAL

See the supplementary material for SEM images of Ni powder (Fig. S1); horizontal cross sectional CT images of the composite (Fig. S2); resistance measurement setup for the developed composites (Fig. S3); repeatability test of stretching and releasing the composites (Fig. S4); comparison of the change in resistance of composites with the addition of ionic liquids (Fig. S5); comparison of the change in resistance of composites with the addition of ionic liquids (Fig. S6); SEM images of the Ag nanowire composite surface W/and W/O (with/and and with/out) strain deformation (Fig. S7); SEM images of the Ni and ionic liquid composite surface W/and W/O strain deformation (Fig. S8); average of breaking strain from five tensile tests (Fig. S9); fabrication method of the balloon-type soft actuator (Fig. S10); fabrication method of the wearable demonstration device (Fig. S11); composite resistance change with elbow bending angle (Fig. S12); Young's modulus of the composites (Table S1); soft actuator demonstration (Video S1); and wearable device demonstration (Video S2).

## Data Availability

The data that support the findings of this study are available within the article and its supplementary material.
